# Polyphyllin II attenuates renal fibrosis in diabetic kidney disease partly through regulation of autophagy and PI3K/AKT/mTOR signaling

**DOI:** 10.3389/fphar.2026.1822990

**Published:** 2026-07-23

**Authors:** Xiaozhu Sun, Xinfang Qin, Huixin Bi

**Affiliations:** 1 Department of Nephrology, The Fifth Affiliated Hospital of Southern Medical University, Guangzhou, Guangdong, China; 2 Department of Nephrology, The Second Affiliated Hospital of Harbin Medical University, Harbin, Heilongjiang, China; 3 Guilin Medical University, Guilin, Guangxi, China

**Keywords:** autophagy, diabetic kidney disease, mesangial cells, PI3K/AKT/mTOR, polyphyllin II, rhizoma paridis

## Abstract

**Background:**

Diabetic kidney disease (DKD) is a leading cause of end-stage renal disease (ESRD) worldwide, and renal fibrosis is a key pathological feature driving disease progression. Polyphyllin II (PPII), a steroidal saponin isolated from Rhizoma Paridis, has shown renoprotective potential; however, its effects and underlying mechanisms in DKD remain unclear.

**Methods:**

Network pharmacology was used to predict the potential targets and pathways of PPII in DKD. Experimental validation was performed in streptozotocin (STZ)-induced DKD mice treated with PPII (8 mg/kg/day for 4 weeks) and in high-glucose (HG)-stimulated glomerular mesangial cells (MCs). Renal function, urinary protein excretion, and histopathological changes were evaluated. Apoptosis was assessed by flow cytometry. Autophagy- and fibrosis-related proteins were examined by Western blotting, and autophagic flux was further evaluated using chloroquine (CQ). Rescue experiments were performed using 3-methyladenine (3-MA). Activation of the phosphoinositide 3-kinase/protein kinase B/mammalian target of rapamycin (PI3K/AKT/mTOR) pathway was assessed by measuring the phosphorylation levels of pathway components.

**Results:**

Network pharmacology identified PI3K/AKT signaling as a potentially important pathway involved in the effects of PPII in DKD. In DKD mice, PPII partially improved renal dysfunction, as reflected by reduced urinary protein excretion, serum creatinine (Scr), and uric acid (UA) levels, as well as increased serum albumin (Alb). Histological analyses showed that PPII attenuated glomerular hypertrophy, mesangial expansion, and renal fibrosis. In HG-stimulated MCs, PPII reduced apoptosis and decreased the expression of fibrosis-related proteins. In both renal tissues and MCs, PPII treatment was associated with reduced p62 expression and an increased LC3-II/LC3-I ratio. CQ-based autophagic flux analysis suggested that PPII may enhance autophagic flux, while 3-MA partially attenuated the anti-fibrotic effects of PPII. In addition, PPII partially reduced HG- and DKD-associated activation of the PI3K/AKT/mTOR pathway.

**Conclusion:**

PPII alleviated renal injury and fibrosis in experimental DKD models and was associated with improved autophagy and reduced fibrotic responses. These effects may be related to modulation of the PI3K/AKT/mTOR signaling pathway. PPII may therefore represent a potential therapeutic candidate for DKD, although further studies are needed to clarify its molecular mechanisms and translational relevance.

## Introduction

Diabetic kidney disease (DKD) is one of the most common and severe microvascular complications of diabetes mellitus and remains a leading cause of end-stage renal disease (ESRD) worldwide (GBD 2021 Risk Factors Collaborators, 2024; Perkovic et al., 2024; Gupta et al., 2023; Agur et al., 2025). Beyond classical renal hemodynamic and metabolic abnormalities, recent evidence indicates that gut microbiota-mediated metabolic reprogramming contributes to the development of host metabolic diseases, suggesting that systemic metabolic and inflammatory regulation may also participate in DKD progression (Wang et al., 2026). With the increasing global prevalence of diabetes, the burden of DKD continues to rise, posing significant clinical and socioeconomic challenges. Although current therapeutic strategies, including glycemic control and renin–angiotensin system blockade, can delay disease progression, many patients still develop progressive renal dysfunction (Rashid et al., 2025; Tanase et al., 2022). Therefore, there is an urgent need to identify novel therapeutic targets and effective interventions for DKD.

Renal fibrosis is a hallmark of DKD and a key determinant of disease progression. It is characterized by excessive accumulation of extracellular matrix (ECM) components, leading to structural damage and loss of renal function. Among the various renal cell types, glomerular mesangial cells (MCs) play a critical role in maintaining glomerular homeostasis. Under hyperglycemic conditions, MCs undergo phenotypic changes, including increased proliferation, enhanced ECM production, and apoptosis, ultimately contributing to glomerulosclerosis and renal fibrosis (Tang et al., 2021; Zhao et al., 2024; Hu et al., 2024; Zhang et al., 2025; Stanigut et al., 2025).

Autophagy is an evolutionarily conserved cellular process that degrades damaged organelles and proteins, thereby maintaining cellular homeostasis. Increasing evidence suggests that impaired autophagy contributes to the development and progression of DKD, whereas restoration of autophagic activity may exert protective effects in renal cells (Stanigut et al., 2025; Liu et al., 2024; Hu et al., 2023; Su et al., 2022; Han et al., 2023). The phosphoinositide 3-kinase/protein kinase B/mammalian target of rapamycin (PI3K/AKT/mTOR) signaling pathway is a central regulator of cell survival, metabolism, and autophagy. Dysregulation of this pathway has been implicated in DKD and is associated with suppressed autophagy and enhanced fibrotic responses (Dong et al., 2023; Li et al., 2025; Tang et al., 2022; Cai et al., 2025; Kong et al., 2023).

Traditional Chinese medicine has been widely used in the treatment of chronic kidney diseases. In particular, medicine-food homology plants and their bioactive constituents have attracted attention as potential interventions for DKD because of their multi-target regulatory properties and relatively favorable safety profiles (Yan et al., 2026). Rhizoma Paridis, a commonly used medicinal herb, has been reported to possess anti-inflammatory, antioxidant, and anti-fibrotic properties. Polyphyllin II (PPII), a major steroidal saponin derived from Rhizoma Paridis, has demonstrated protective effects in various disease models, including renal injury (Li et al., 2022; Jiang et al., 2024; Miao et al., 2024; Jiao et al., 2022). However, its role and underlying mechanisms in DKD remain incompletely understood.

In the present study, we investigated the effects of PPII on renal injury and fibrosis in both *in vivo* and *in vitro* models of DKD. Furthermore, we explored whether these effects are associated with modulation of autophagy and regulation of the PI3K/AKT/mTOR signaling pathway.

## Materials and methods

### Chemicals and reagents

Polyphyllin II (PPII) was purchased from Vekochi Biotechnology Co., Ltd (Sichuan, China). Streptozotocin (STZ), sodium citrate buffer, 4% paraformaldehyde, bicinchoninic acid (BCA) protein assay kit, hematoxylin and eosin (HE), periodic acid–Schiff (PAS), and Masson’s trichrome staining kits were obtained from Solarbio Bioscience and Technology Co., Ltd (Beijing, China). Fetal bovine serum (FBS) was also purchased from Solarbio.

Primary antibodies against p62, microtubule-associated protein 1 light chain 3B (LC3B), collagen type I alpha 1 (COL1A1), fibronectin 1 (FN1), PI3K, AKT, mTOR, phosphorylated PI3K (p-PI3K), phosphorylated AKT (p-AKT), phosphorylated mTOR (p-mTOR), and β-actin were obtained from ABclonal (Wuhan, China) and Proteintech (Wuhan, China).

### Network pharmacology analysis

**FIGURE 1:**
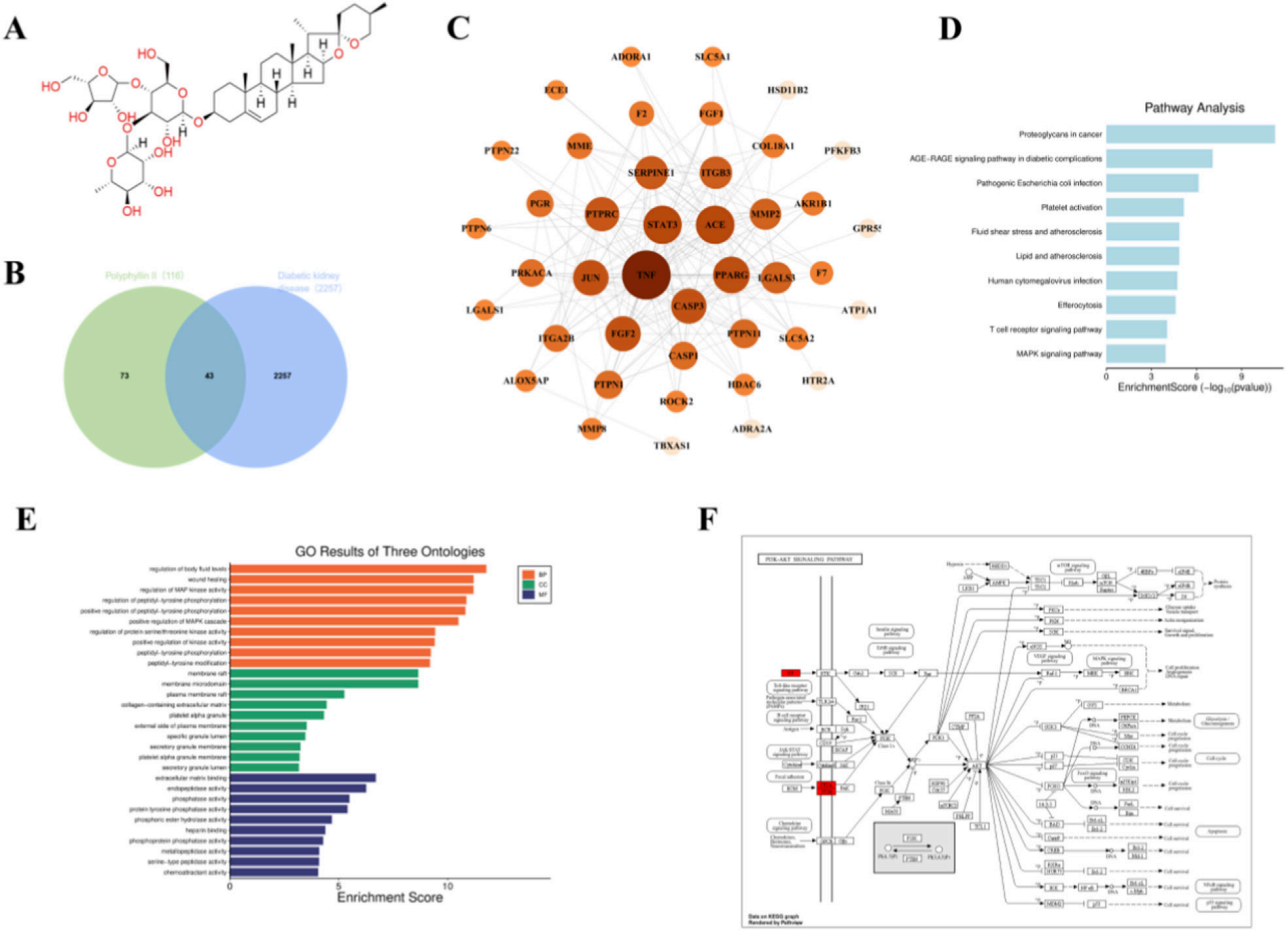
Network Pharmacology Analysis **(A)** Chemical structure of PPII **(B)** Identification of overlapping targets between PPII and DKD **(C)** Protein–protein interaction (PPI) network of shared targets **(D)** KEGG pathway enrichment analysis **(E)** GO functional enrichment analysis **(F)** PI3K/AKT signaling pathway diagram with PPII-associated targets highlighted in red.

The chemical structure and Simplified Molecular Input Line Entry System (SMILES) information of Polyphyllin II (PPII) were obtained from the PubChem database (Figure 1A). Potential targets of PPII were predicted using the SwissTargetPrediction database, while diabetic kidney disease (DKD)-related targets were collected from the GeneCards and Online Mendelian Inheritance in Man (OMIM) databases. Common targets between PPII and DKD were identified using an online Venn diagram tool. Protein–protein interaction (PPI) networks were constructed using the Search Tool for the Retrieval of Interacting Genes/Proteins (STRING) database and visualized using Cytoscape software. Gene Ontology (GO) functional annotation and Kyoto Encyclopedia of Genes and Genomes (KEGG) pathway enrichment analyses were performed using an online bioinformatics platform (Liu et al., 2025; Liu et al., 2023).

### Animal experiments

Male C57BL/6J mice (6–8 weeks old, 20 ± 2 g) were obtained from Hunan Jingda 119 Biotechnology Co., Ltd. and maintained under specific pathogen-free (SPF) conditions with a 12-h light/dark cycle and *ad libitum* access to food and water. All experimental procedures were approved by the Ethics Committee of Guilin Medical University. Mice were allowed to acclimate for 1 week prior to the start of experiments. Diabetes was induced at week 0 by intraperitoneal injection of streptozotocin (STZ, 50 mg/kg/day) for five consecutive days, while control mice received an equivalent volume of citrate buffer. One week after STZ injection, fasting blood glucose (FBG) was measured, and mice with FBG ≥11.1 mmol/L were considered diabetic. The diabetic kidney disease (DKD) model was allowed to develop over 6 weeks, and successful establishment was confirmed when 24-h urinary total protein (24-UTP) excretion increased tenfold relative to controls (Azushima et al., 2018). At week 7, mice were randomly assigned to four groups (n = 8 per group): control (CON), DKD, control + Polyphyllin II (CON + PPII), and DKD + Polyphyllin II (DKD + PPII). Polyphyllin II (PPII) was administered via oral gavage at 8 mg/kg/day for 4 weeks, while control mice received an equivalent volume of distilled water. The dose of PPII was selected based on previously reported oral/intragastric dosing studies and our preliminary dose-ranging experiment. Since PPII was administered by oral gavage in this study, we referred to a previous mouse study in which Paris Saponin II was given orally at 20 mg/kg/day (Chen et al., 2019). Considering the repeated treatment required in the DKD model and the mechanistic focus of the present study, 16 mg/kg/day was set as a relatively high exploratory dose below the reported oral dose, and 8 and 4 mg/kg/day were selected using a two-fold dose de-escalation design. In our preliminary experiment using C57BL/6J DKD mice, 4 mg/kg/day showed detectable protective effects, 8 mg/kg/day produced more stable and robust improvements in DKD-related renal endpoints, whereas 16 mg/kg/day did not provide obvious additional benefit. Therefore, 8 mg/kg/day was selected as a representative effective dose for subsequent mechanistic studies, balancing efficacy, reproducibility, and avoidance of unnecessary high-dose exposure. At week 11, all mice were sacrificed, and tissue and urine samples were collected for downstream analyses.

### Biochemical measurements

Blood glucose was measured using a glucometer. Twenty-four-hour urine samples were collected using metabolic cages. Urinary protein concentration, serum creatinine (Scr), blood urea nitrogen (BUN), uric acid (UA), and albumin (Alb) were measured using an automatic biochemical analyzer. The urinary albumin-to-creatinine ratio (UACR) was calculated.

### Histological analysis

Kidney tissues were fixed in 4% paraformaldehyde, embedded in paraffin, and sectioned at 4 μm. Sections were stained with HE, PAS, and Masson’s trichrome staining kits according to the manufacturer’s instructions. Histopathological changes were observed under a light microscope.

### Cell culture and treatment

Glomerular mesangial cells (MCs) were kindly provided by Prof. Xiaoxi Zhang (Department of Nephrology, Guilin Medical University, China) and cultured in Dulbecco’s modified Eagle’s medium (DMEM) containing 5.5 mM glucose (normal glucose, NC), supplemented with 10% fetal bovine serum (FBS) and 1% penicillin/streptomycin. Cells were maintained at 37 °C in a humidified incubator with 5% CO_2_ and passaged at approximately 70%–80% confluence. To mimic diabetic conditions *in vitro*, MCs were exposed to high-glucose (HG) medium containing 25 mM glucose. To control for osmotic effects in the apoptosis assay, a mannitol osmotic control (MNT) group was included. Cells in the MNT group were cultured in normal-glucose medium containing 5.5 mM glucose supplemented with 19.5 mM mannitol for the same duration as the HG treatment.

For PPII treatment, MCs were incubated with PPII (10 μM) for 24 h under HG conditions. The concentration of PPII was selected based on preliminary dose–response experiments, which showed no significant cytotoxicity while maintaining measurable biological activity, and was also consistent with concentrations reported in previous studies. For autophagic flux analysis, chloroquine (CQ; 10 μM), a lysosomal inhibitor, was added 4 h before cell collection to block autophagosome degradation. The experimental groups were as follows: NC, HG, HG + PPII, HG + CQ, and HG + PPII + CQ. For rescue experiments, 3-methyladenine (3-MA; 5 mM), an autophagy inhibitor, was used. Cells were pretreated with 3-MA for 2 h, followed by PPII treatment under HG conditions. The experimental groups included NC, HG, HG + PPII, and HG + PPII + 3-MA. After treatment, cells were harvested for transmission electron microscopy, flow cytometry, and Western blot analysis.

### Apoptosis analysis

Cell apoptosis was assessed using Annexin V-FITC/propidium iodide (PI) staining followed by flow cytometry according to the manufacturer’s protocol.

### Transmission electron microscopy (TEM)

Mesangial cells were fixed with 2.5% glutaraldehyde at 4 °C overnight and post-fixed with 1% osmium tetroxide. Samples were dehydrated through a graded ethanol series, embedded in epoxy resin, and ultrathin sections (70 nm) were prepared. Sections were stained with uranyl acetate and lead citrate and observed using a transmission electron microscope (JEOL JEM-1400) at 80 kV to evaluate mitochondrial morphology and autophagosome formation.

### Western blot analysis

Western blot (WB) analysis was performed as follows. Total proteins from kidney tissues and mesangial cells were extracted using radioimmunoprecipitation assay (RIPA) lysis buffer containing protease and phosphatase inhibitors. Protein concentrations were determined using a BCA protein assay kit according to the manufacturer’s instructions. Equal amounts of protein (20–40 μg per sample) were separated by sodium dodecyl sulfate–polyacrylamide gel electrophoresis (SDS-PAGE) and transferred onto polyvinylidene fluoride (PVDF) membranes. Membranes were blocked with 5% skim milk in Tris-buffered saline with Tween 20 (TBST) at room temperature for 1 h and then incubated overnight at 4 °C with primary antibodies against fibronectin 1 (FN1), collagen type I alpha 1 (COL1A1), p62, LC3B, PI3K, phosphorylated PI3K (p-PI3K), AKT, phosphorylated AKT (p-AKT), mTOR, phosphorylated mTOR (p-mTOR), and β-actin; the dilution of p62 antibody was 1:20,000, LC3B was diluted at 1:2,000, and all other primary antibodies were used at a dilution of 1:1,000. After washing with TBST, membranes were incubated with horseradish peroxidase (HRP)-conjugated secondary antibodies (1:5,000 dilution) for 1 h at room temperature. Protein bands were visualized using enhanced chemiluminescence (ECL) reagents and detected using a chemiluminescence imaging system. Band intensities were quantified using ImageJ software and normalized to β-actin as the internal loading control.

### Statistical analysis

All data are presented as mean ± standard error of the mean (SEM). Statistical analyses were performed using SPSS 26.0. Normality and homogeneity of variance were assessed before parametric analysis. For time-course data, including body weight, fasting blood glucose, and 24-h urinary total protein, two-way repeated-measures ANOVA followed by *post hoc* multiple-comparison tests was used. For comparisons among multiple groups at a single time point, one-way ANOVA followed by appropriate *post hoc* tests was performed. Histological quantification and Western blot densitometric analyses were performed in a blinded manner where applicable. A value of P < 0.05 was considered statistically significant.

## Results

### Network pharmacology analysis

A total of 116 PPII-related targets were identified. For DKD, 2,107 targets were initially retrieved from the GeneCards database, and after integrating 247 targets from the OMIM database, a total of 2,300 DKD-related targets were obtained. By intersecting PPII-related targets with DKD-related targets, 43 common targets were identified (Figure 1B). The protein–protein interaction (PPI) data were imported into Cytoscape for network construction and topological analysis, in which key parameters including degree, betweenness centrality, and closeness centrality were calculated, and the top 10 nodes with the highest degree values were defined as core targets. The PPI network consisted of 42 nodes and 174 edges, with an average degree of 8.286, and node size and color were adjusted based on degree values, with larger sizes and darker colors indicating stronger interactions with other targets (Figure 1C). KEGG pathway enrichment analysis revealed that the PI3K/AKT signaling pathway was among the most significantly enriched pathways (Figure 1D), and the pathway diagram highlighted potential therapeutic targets of PPII, with key nodes indicated in red (Figure 1F). GO enrichment analysis of the overlapping targets was subsequently performed, including biological process (BP), cellular component (CC), and molecular function (MF) categories. Terms with P < 0.05 were considered statistically significant, resulting in 1,472 BP terms, 66 CC terms, and 148 MF terms (Figure 1E).

### PPII improves renal dysfunction in DKD mice

**FIGURE 2:**
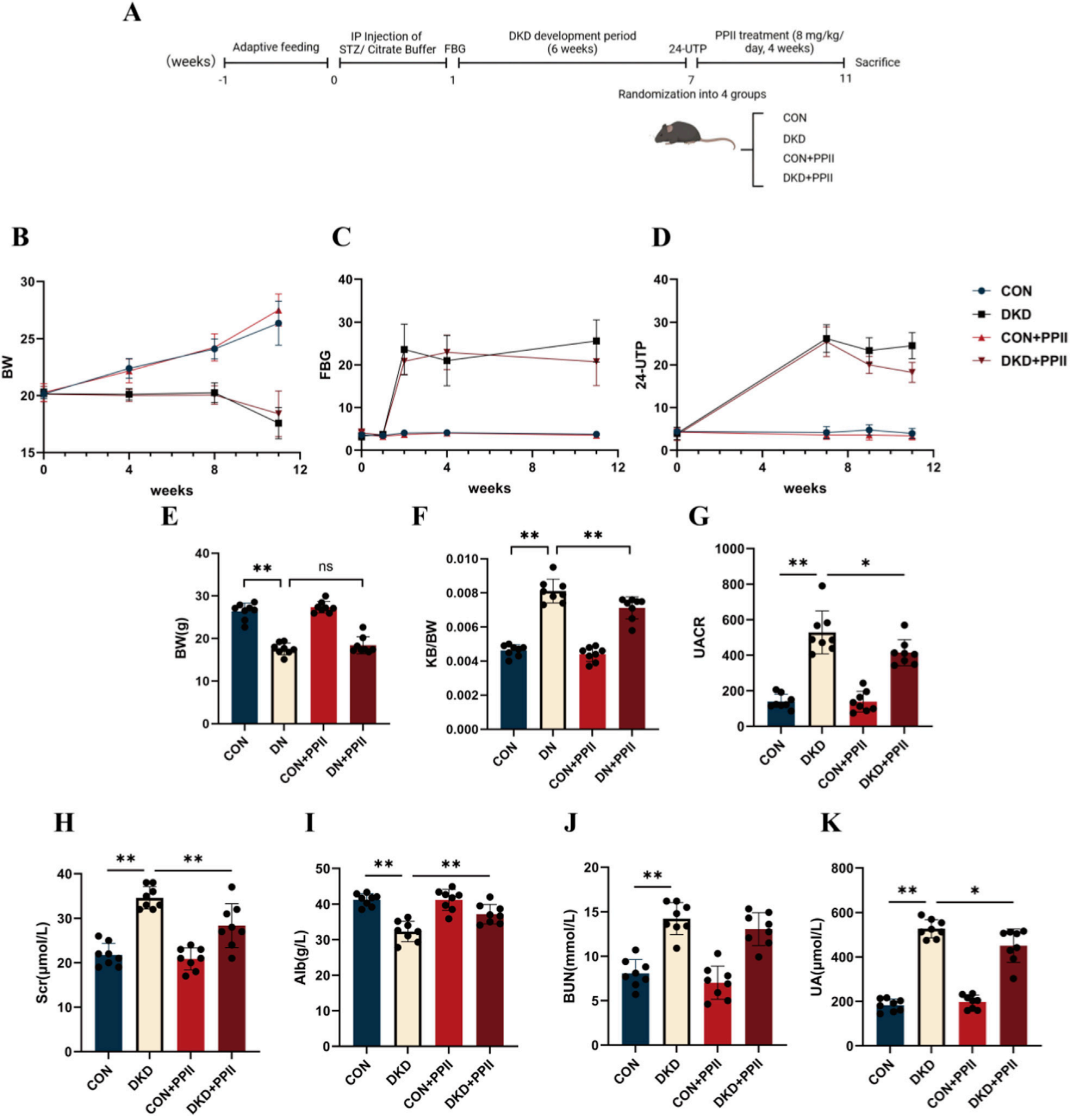
PPII Improves Renal Dysfunction in DKD Mice **(A)** Schematic diagram of the experimental design and treatment protocol. Mice were assigned to CON, DKD, CON + PPII, and DKD + PPII groups. STZ was used to induce DKD, and PPII was administered by oral gavage during the intervention period **(B–D)** Time-course analysis of body weight (BW) **(B)**, fasting blood glucose (FBG) **(C)**, and 24-h urinary total protein (24-UTP) **(D)** in the indicated groups during the experimental period **(E–K)** Quantitative analysis of final BW **(E)**, kidney weight/body weight ratio (KW/BW) **(F)**, urinary albumin-to-creatinine ratio (UACR) **(G)**, serum creatinine (Scr) **(H)**, serum albumin (Alb) **(I)**, blood urea nitrogen (BUN) **(J)**, and uric acid (UA) **(K)**. Data are presented as mean ± SEM (n = 8 per group). Statistical significance was determined as described in the Statistical Analysis section. *P < 0.05, **P < 0.01; ns, not significant.

As shown in the experimental scheme (Figure 2A), DKD mice exhibited progressive BW loss during the study period (Figure 2B), and the final BW was also reduced at the end of the experiment (Figure 2E). PPII treatment partially attenuated these changes. FBG remained markedly elevated in DKD mice, and PPII treatment showed a modest trend toward improvement, although hyperglycemia was not fully reversed (Figure 2C). In addition, 24-UTP was markedly increased in DKD mice, whereas PPII treatment reduced urinary protein excretion (Figure 2D). Further analysis of renal function-related parameters showed that DKD mice exhibited significantly increased KW/BW, UACR, Scr, BUN, and UA, accompanied by decreased Alb levels (Figures 2F–K). PPII treatment partially improved these abnormalities, as evidenced by reduced UACR, Scr, and UA levels and increased Alb levels (Figures 2G–I,K), while BUN showed a decreasing trend (Figure 2J). No significant differences were observed between the CON and CON + PPII groups in these parameters, suggesting that PPII was well tolerated under normal conditions.

### PPII improves renal histopathological lesions in DKD mice

**FIGURE 3:**
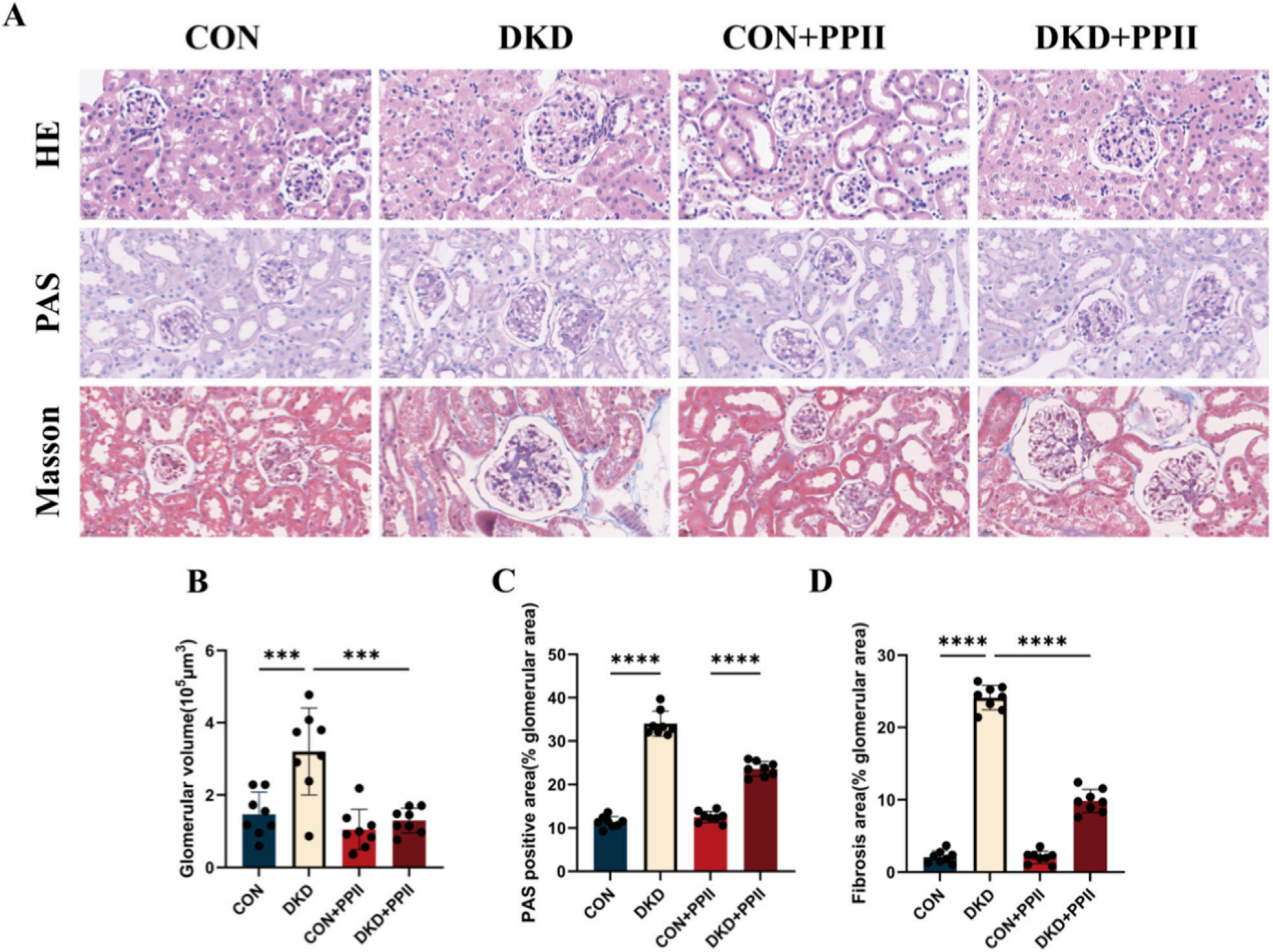
PPII Improves Renal Histopathological Lesions in DKD Mice **(A)** Representative images of kidney sections stained with hematoxylin and eosin (HE), periodic acid–Schiff (PAS), and Masson’s trichrome in the CON, DKD, CON + PPII, and DKD + PPII groups. HE staining shows general renal morphology, PAS staining indicates mesangial matrix expansion, and Masson’s trichrome staining reflects collagen deposition and fibrosis **(B–D)** Quantitative analysis of glomerular volume **(B)**, PAS-positive area (% glomerular area) **(C)**, and fibrosis area (% glomerular area) based on Masson’s trichrome staining **(D)**. Data are presented as mean ± SEM (n = 8 per group). Statistical significance was determined as described in the Statistical Analysis section. ***P < 0.001, ****P < 0.0001.

Histological analysis revealed marked renal structural damage in DKD mice. HE staining showed that DKD mice exhibited glomerular hypertrophy and thickening of the glomerular basement membrane, which were partially ameliorated by PPII treatment (Figures 3A,B). PAS staining demonstrated a significant increase in mesangial matrix expansion in DKD mice, whereas PPII treatment reduced the PAS-positive area in the glomeruli (Figures 3A,C). In addition, Masson’s trichrome staining revealed excessive collagen deposition and fibrosis in DKD mice, which were markedly attenuated following PPII administration (Figures 3A,D). Quantitative analysis further confirmed that glomerular volume, PAS-positive area, and fibrosis area were significantly increased in DKD mice, while PPII treatment effectively reduced these pathological changes (Figures 3B–D).

### PPII alleviates MC injury and is associated with improved autophagy and reduced fibrosis *In Vitro* and *In Vivo*

**FIGURE 4:**
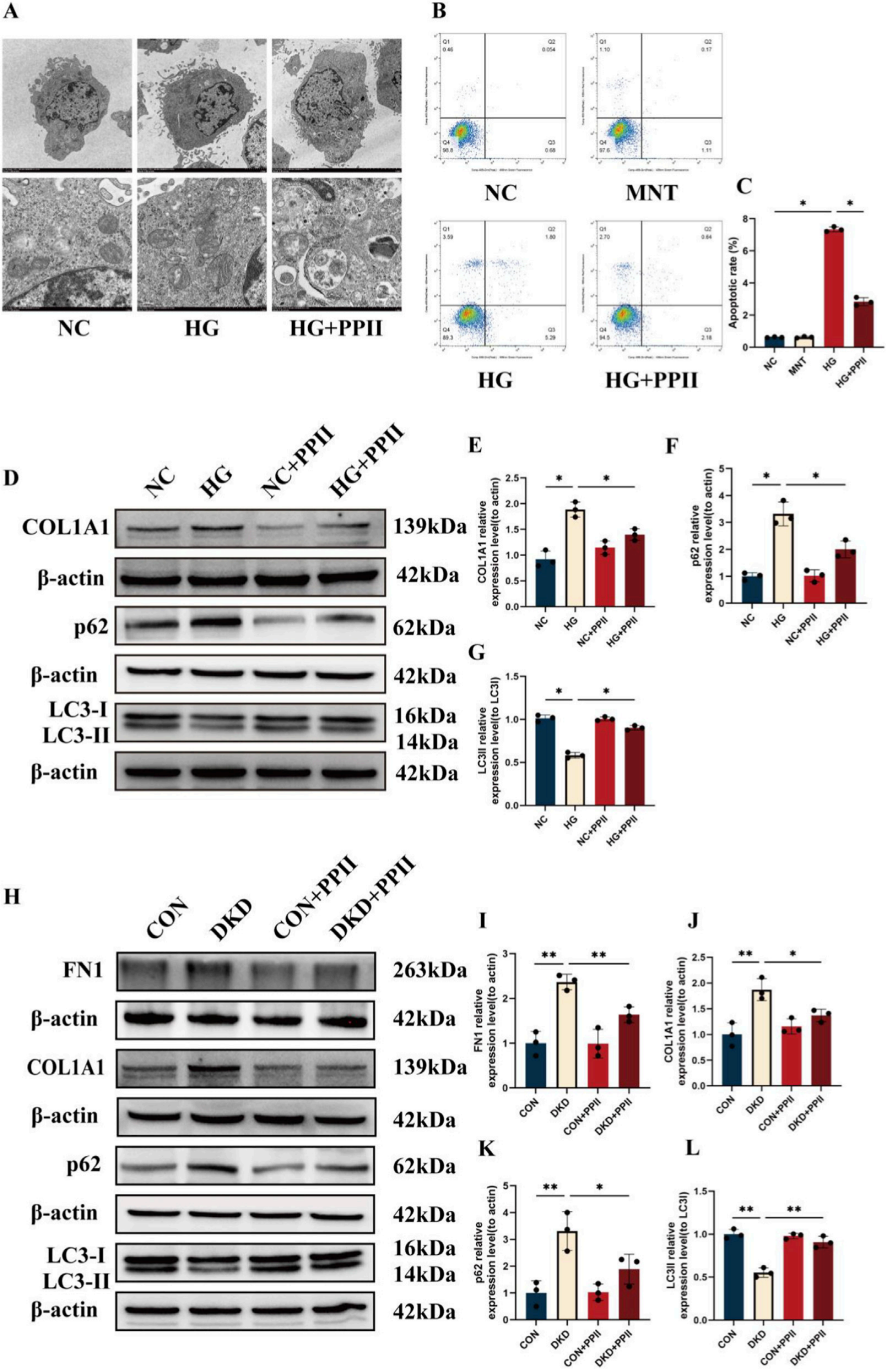
PPII Alleviates MC Injury and Is Associated with Improved Autophagy and Reduced Fibrosis *In Vitro* and *In Vivo* **(A)** Representative TEM images showing ultrastructural changes and autophagosome formation in MCs under NC, HG, and HG + PPII conditions **(B)** Representative Annexin V-FITC/PI flow cytometry plots of apoptosis in MCs under NC, MNT, HG, and HG + PPII conditions **(C)** Quantification of apoptotic rate based on flow cytometry analysis **(D)** Representative WB images showing the expression of COL1A1, p62, and LC3 (LC3-I and LC3-II) in MCs under NC, HG, NC + PPII, and HG + PPII conditions, with β-actin as the loading control **(E–G)** Densitometric analysis of COL1A1 **(E)** and p62 **(F)**, normalized to β-actin, and the LC3-II/LC3-I ratio **(G) (H)** Representative WB images showing the expression of FN1, COL1A1, p62, and LC3 (LC3-I and LC3-II) in renal tissues from CON, DKD, CON + PPII, and DKD + PPII groups, with β-actin as the loading control **(I–L)** Densitometric analysis of FN1 **(I)**, COL1A1 **(J)**, and p62 **(K)**, normalized to β-actin, and the LC3-II/LC3-I ratio **(L)**. Data are presented as mean ± SEM. For *in vitro* assays, n = 3 independent experiments. For *in vivo* WB analyses, n = 3 biological samples per group. Statistical significance was determined as described in the Statistical Analysis section. *P < 0.05, **P < 0.01.

HG stimulation markedly induced cellular injury in MCs. TEM revealed mitochondrial swelling, disrupted mitochondrial membrane structure, and matrix rarefaction in the HG group compared with the NC group. In contrast, PPII treatment partially restored mitochondrial integrity and increased the number of autophagosomes, suggesting enhanced autophagic activity (Figure 4A). Flow cytometry analysis further demonstrated that HG exposure significantly increased the apoptotic rate of MCs, whereas PPII treatment reduced HG-induced apoptosis (Figures 4B,C).

Consistently, HG stimulation was associated with enhanced fibrotic responses and impaired autophagy in MCs. Western blot analysis showed that HG increased COL1A1 and p62 expression while decreasing the LC3-II/LC3-I ratio (Figure 4D). Densitometric analysis confirmed that COL1A1 and p62 levels were significantly elevated under HG conditions (Figures 4E,F), whereas the LC3-II/LC3-I ratio was reduced (Figure 4G). PPII treatment partially reversed these changes, as reflected by decreased COL1A1 and p62 expression and an increased LC3-II/LC3-I ratio (Figures 4E–G).

*In vivo*, similar trends were observed in DKD mice. Western blot analysis showed that DKD increased the expression of fibrosis-related proteins FN1 and COL1A1, along with increased p62 expression and a reduced LC3-II/LC3-I ratio in renal tissues (Figure 4H). Quantitative analysis further demonstrated that FN1, COL1A1, and p62 levels were significantly elevated in the DKD group (Figures 4I–K), whereas the LC3-II/LC3-I ratio was decreased (Figure 4L). PPII treatment partially improved these alterations, as evidenced by reduced FN1, COL1A1, and p62 expression and an increased LC3-II/LC3-I ratio in kidney tissues (Figures 4I–L). Taken together, these results suggest that PPII alleviates HG-induced MC injury and is associated with improved autophagy and reduced fibrotic responses in both MCs and renal tissues from DKD mice.

### PPII promotes autophagic flux and its anti-fibrotic effects are associated with autophagy in MCs

**FIGURE 5:**
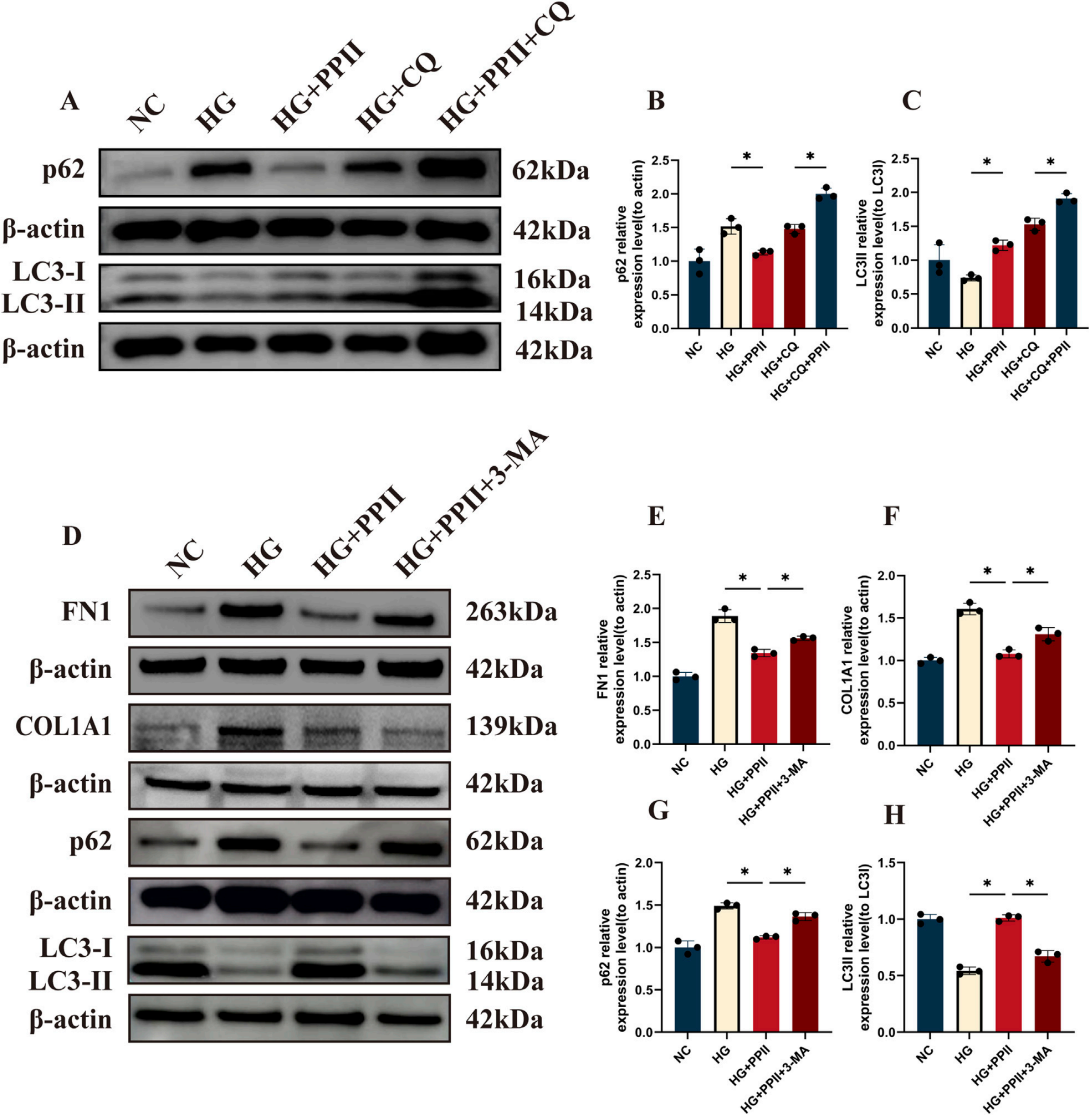
PPII Promotes Autophagic Flux and Its Anti-fibrotic Effects Are Associated with Autophagy in MCs **(A)** Representative WB images showing the expression of p62 and LC3 (LC3-I and LC3-II) in MCs under NC, HG, HG + PPII, HG + CQ, and HG + PPII + CQ conditions, with β-actin as the loading control **(B,C)** Densitometric analysis of p62 expression **(B)**, normalized to β-actin, and the LC3-II/LC3-I ratio **(C) (D)** Representative WB images showing the expression of FN1, COL1A1, p62, and LC3 (LC3-I and LC3-II) in MCs under NC, HG, HG + PPII, and HG + PPII + 3-MA conditions, with β-actin as the loading control **(E–H)** Densitometric analysis of FN1 **(E)**, COL1A1 **(F)**, and p62 **(G)**, normalized to β-actin, and the LC3-II/LC3-I ratio **(H)**. CQ was used to inhibit autophagosome degradation to assess autophagic flux, whereas 3-MA was used to inhibit autophagy. Data are presented as mean ± SEM from three independent experiments (n = 3). Statistical significance was determined as described in the Statistical Analysis section. *P < 0.05, **P < 0.01.

To further investigate whether PPII modulates autophagic flux in MCs, CQ was used to block autophagosome degradation. As shown in Figure 5A, HG stimulation increased p62 expression and decreased the LC3-II/LC3-I ratio compared with the control group, indicating impaired autophagy. PPII treatment partially reversed these changes, as reflected by reduced p62 levels (Figure 5B) and an increased LC3-II/LC3-I ratio (Figure 5C). CQ treatment led to a marked accumulation of LC3-II under HG conditions, confirming effective inhibition of autophagic degradation. Notably, co-treatment with PPII and CQ resulted in a further increase in LC3-II levels compared with CQ alone, suggesting that PPII may enhance autophagic flux rather than simply inducing autophagosome accumulation (Figures 5A–C).

To examine whether the protective effects of PPII are associated with autophagy, 3-MA was applied. As shown in Figure 5D, HG stimulation significantly increased the expression of fibrosis-related proteins FN1 and COL1A1, whereas PPII treatment reduced their expression. Co-treatment with 3-MA partially attenuated the inhibitory effects of PPII on FN1 (Figure 5E) and COL1A1 (Figure 5F). In addition, the PPII-induced decrease in p62 (Figure 5G) and increase in the LC3-II/LC3-I ratio (Figure 5H) were also partially reversed by 3-MA. Taken together, these findings suggest that PPII promotes autophagic flux in MCs and that its anti-fibrotic effects are, at least in part, associated with regulation of autophagy.

### PPII is associated with inhibition of PI3K/AKT/mtor pathway activation in MCs and DKD mice

**FIGURE 6:**
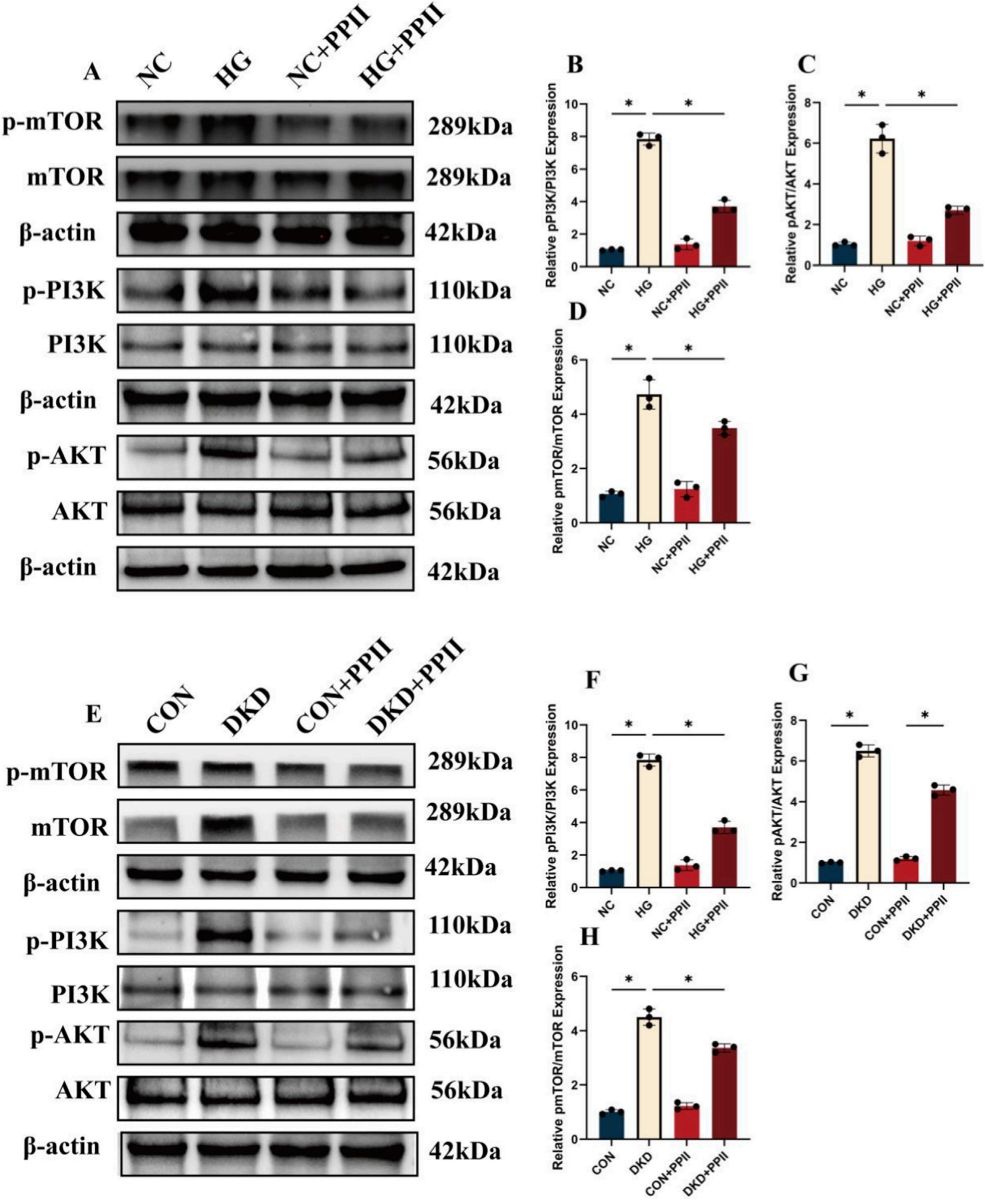
PPII Is Associated with Inhibition of PI3K/AKT/mTOR Pathway Activation in MCs and DKD Mice **(A)** Representative WB images showing the expression of phosphorylated and total PI3K, AKT, and mTOR in MCs under NC, HG, NC + PPII, and HG + PPII conditions, with β-actin as the loading control **(B–D)** Densitometric analysis of the phosphorylation ratios p-PI3K/PI3K **(B)**, p-AKT/AKT **(C)**, and p-mTOR/mTOR **(D)** in MCs **(E)** Representative WB images showing the expression of phosphorylated and total PI3K, AKT, and mTOR in renal tissues from CON, DKD, CON + PPII, and DKD + PPII groups, with β-actin as the loading control **(F–H)** Densitometric analysis of the phosphorylation ratios p-PI3K/PI3K **(F)**, p-AKT/AKT **(G)**, and p-mTOR/mTOR **(H)** in renal tissues. Phosphorylation levels were expressed as the ratios of phosphorylated proteins to their corresponding total proteins. Data are presented as mean ± SEM. For *in vitro* assays, n = 3 independent experiments. For *in vivo* WB analyses, n = 3 biological samples per group. Statistical significance was determined as described in the Statistical Analysis section. *P < 0.05, **P < 0.01.

To investigate whether PPII regulates the PI3K/AKT/mTOR signaling pathway, phosphorylation levels of key pathway components were assessed by Western blot analysis. In MCs, HG stimulation markedly increased phosphorylation of PI3K, AKT, and mTOR compared with the NC group, indicating activation of the PI3K/AKT/mTOR pathway (Figure 6A). Densitometric analysis confirmed that the phosphorylation ratios p-PI3K/PI3K, p-AKT/AKT, and p-mTOR/mTOR were significantly elevated under HG conditions (Figures 6B–D). PPII treatment partially attenuated HG-induced pathway activation, as reflected by reduced phosphorylation of PI3K, AKT, and mTOR (Figures 6A–D). No significant differences were observed between the NC and NC + PPII groups, suggesting that PPII had minimal effects on basal pathway activity.

Consistent results were observed *in vivo*. In renal tissues from DKD mice, phosphorylation of PI3K, AKT, and mTOR was markedly increased compared with the control group (Figure 6E). Quantitative analysis further demonstrated that the phosphorylation ratios p-PI3K/PI3K, p-AKT/AKT, and p-mTOR/mTOR were significantly elevated in the DKD group (Figures 6F–H). PPII treatment partially reversed these changes, indicating that PPII is associated with suppression of PI3K/AKT/mTOR pathway activation in DKD mice. Taken together, these findings suggest that the protective effects of PPII in DKD may be associated with modulation of the PI3K/AKT/mTOR signaling pathway.

## Discussion

DKD is a leading cause of ESRD worldwide, and progressive renal fibrosis remains a key driver of irreversible functional decline (Gupta et al., 2023; Hu et al., 2023; Liu et al., 2022; Chen et al., 2022). In this study, we provide evidence that PPII mitigates DKD-associated renal injury and fibrotic remodeling, and that these protective effects are accompanied by restoration of autophagy and attenuation of PI3K/AKT/mTOR pathway activation. Collectively, our findings support the concept that targeting autophagy-related dysregulation may represent a viable strategy to slow fibrotic progression in DKD.

A major strength of the present work is the consistency between *in vivo* and *in vitro* observations. In STZ-induced DKD mice, PPII improved renal functional parameters and alleviated histopathological lesions indicative of glomerular hypertrophy, mesangial expansion, and collagen deposition, suggesting an overall renoprotective profile. In parallel, PPII reduced HG-induced injury in MCs, including apoptosis and fibrotic protein expression. These concordant results across experimental systems reinforce the robustness of the observed anti-fibrotic effects and indicate that MCs may represent an important cellular target contributing to PPII-mediated renoprotection.

Mechanistically, our data suggest that autophagy is involved in the protective actions of PPII. Autophagy impairment has been increasingly recognized as a contributor to DKD progression, particularly in the context of sustained metabolic stress and ECM accumulation (Stanigut et al., 2025; Liu et al., 2024; Hu et al., 2023; Su et al., 2022; Han et al., 2023). In our models, DKD/HG conditions were associated with increased p62 and decreased LC3-II/LC3-I ratios, consistent with suppressed autophagic activity. Notably, PPII reversed these changes in both renal tissues and MCs. Moreover, CQ-based autophagic flux assessment indicated that PPII likely enhances autophagic flux rather than merely promoting autophagosome accumulation. The observation that 3-MA partially blunted the anti-fibrotic effects of PPII further supports the notion that autophagy contributes, at least in part, to the downstream attenuation of fibrotic responses. Taken together, these findings place autophagy restoration as a plausible mechanistic link between PPII exposure and reduced renal fibrogenesis.

The PI3K/AKT/mTOR pathway is a central upstream regulator of autophagy and is frequently hyperactivated under diabetic conditions, thereby suppressing autophagy and facilitating pro-fibrotic remodeling (Dong et al., 2023; Li et al., 2025; Tang et al., 2022; Cai et al., 2025; Kong et al., 2023). In the present study, DKD/HG stimulation increased phosphorylation of PI3K, AKT, and mTOR, whereas PPII partially reduced pathway activation in both MCs and renal tissues. These results suggest that modulation of PI3K/AKT/mTOR signaling may contribute to PPII-associated restoration of autophagy and suppression of fibrotic responses. Importantly, while the observed correlation is consistent with an autophagy-dependent mechanism, further work will be needed to establish causality and to determine whether PI3K/AKT/mTOR is the primary pathway through which PPII acts or one component of a broader regulatory network.

The selection of the PI3K/AKT/mTOR pathway for experimental validation was supported by the network pharmacology results and by its established role in DKD, autophagy regulation, and fibrotic remodeling (Dong et al., 2023; Li et al., 2025; Tang et al., 2022; Cai et al., 2025; Kong et al., 2023). Although multiple DKD-related targets and pathways were predicted, PI3K/AKT/mTOR signaling provided a biologically coherent link between the predicted network, autophagy impairment, and the anti-fibrotic phenotype observed in this study. Nevertheless, this pathway is unlikely to act in isolation. PI3K/AKT/mTOR signaling may interact with other DKD-related pathways, including AMPK, NF-κB, TGF-β/Smad, and oxidative stress-related signaling, thereby collectively regulating autophagy, inflammation, extracellular matrix accumulation, and renal fibrosis (Shi et al., 2025; Kume and Koya, 2015). Therefore, PPII may exert renoprotective effects through a broader regulatory network rather than a single linear pathway.

Beyond kidney-intrinsic signaling pathways, systemic metabolic regulation may also contribute to DKD progression and the potential actions of PPII. Gut microbiota-mediated metabolic reprogramming has been reported to influence host metabolic homeostasis, inflammatory responses, and downstream signaling pathways involved in metabolic diseases (Wang et al., 2026). Therefore, PPII may potentially affect DKD progression not only through direct regulation of renal signaling pathways, but also through gut-kidney metabolic interactions, although this hypothesis remains to be validated. In addition, the present findings are consistent with the growing interest in medicine-food homology plants and natural bioactive components for DKD intervention, which may offer multi-target regulatory potential by modulating metabolic stress, inflammation, oxidative injury, autophagy, and fibrosis-related pathways (Yan et al., 2026).

Several limitations should be acknowledged. First, although CQ and 3-MA were used to assess the involvement of autophagy, these pharmacological tools may have off-target effects; therefore, genetic approaches such as ATG knockdown/knockout or pathway-specific modulation are needed to further confirm causality. Second, a positive drug control group, such as enalapril or metformin, was not included, which limits direct comparison between PPII and clinically used DKD therapies. Third, inflammatory cytokines in renal tissues were not measured, and the effects of PPII on other renal cell types, including podocytes and tubular epithelial cells, remain unclear. Finally, although network pharmacology suggested multiple potential targets and pathways, this study mainly focused on PI3K/AKT/mTOR signaling, and molecular docking or molecular dynamics simulations were not performed to validate the direct binding mode between PPII and key pathway proteins. These issues should be addressed in future studies.

In summary, our findings indicate that PPII alleviates renal injury and fibrosis in experimental DKD, accompanied by enhanced autophagic activity and reduced PI3K/AKT/mTOR pathway activation. These results suggest that restoration of autophagy may contribute to the anti-fibrotic effects of PPII. However, PPII may act through a broader regulatory network involving autophagy, inflammation, oxidative stress, metabolic remodeling, and fibrotic signaling. Future studies incorporating positive drug controls, genetic validation, molecular docking or molecular dynamics simulations, microbiome-metabolomics analysis, and clinically relevant DKD models will be necessary to further define the therapeutic potential and molecular mechanisms of PPII.

## Conclusion

In conclusion, the present study demonstrates that PPII exerts renoprotective effects in experimental models of DKD. PPII ameliorated renal injury and fibrosis, accompanied by enhanced autophagic activity and reduced activation of the PI3K/AKT/mTOR signaling pathway. These findings suggest that restoration of autophagy may contribute to the protective effects of PPII against DKD-associated fibrotic remodeling. Although further studies incorporating positive drug controls, direct target validation, and broader pathway analysis are required, our results support PPII as a promising therapeutic candidate for DKD management.

## Data Availability

The original contributions presented in the study are included in the article. Further inquiries can be directed to the corresponding author.
